# Metabolic-Brain Functional Coupling Mechanism Underlying iTBS Treatment for Adolescent Depression

**DOI:** 10.31083/AP48591

**Published:** 2026-08-06

**Authors:** Zeyang Zhao, Tao Leng, Peiying Li, Fangyuan Wu, Ling Sun, Bin Zhang

**Affiliations:** ^1^Department of Psychology, Chengde Medical University, 067000 Chengde, Hebei, China; ^2^Institute of Mental Health, Tianjin Anding Hospital, Mental Health Center of Tianjin University, 300222 Tianjin, China; ^3^Institute of Mental Health, Tianjin Anding Hospital, Mental Health Center of Tianjin Medical University, 300222 Tianjin, China

**Keywords:** depression, adolescent, metabolomics, transcranial magnetic stimulation, magnetic resonance imaging

## Abstract

**Background:**

Adolescents with depression pose a significant public health challenge, yet individual variability in treatment response to accelerated intermittent theta-burst stimulation (iTBS) for depression remains substantial. This study aims to integrate metabolomics with multimodal neuroimaging techniques to identify objective biomarkers of response to iTBS treatment and investigate the relationship between brain and metabolism.

**Methods:**

Patients received an accelerated iTBS protocol targeting the left dorsolateral prefrontal cortex. Assessments included plasma metabolomic profiling, resting-state and naturalistic functional magnetic resonance imaging, and clinical scale evaluations conducted at baseline and post-treatment.

**Results:**

The study enrolled 85 adolescents with depression and 48 healthy controls. 53 adolescent patients with depression completed the full course of iTBS treatment and provided blood samples for biomarker detection and analysis. Seventeen differential metabolites were identified that distinguished patients from controls and changed significantly after iTBS treatment. The area under the curve (AUC) for the metabolites 3-hydroxymethylglutaric acid, malonic acid, N-acetylphenylalanine, norepinephrine and tryptophanamide exceeds 0.8. The metabolites isovalerylcarnitine, taurochenodeoxycholic acid, taurodeoxycholic acid, and valerylcarnitine were significantly correlated with the reduction in scores on the Hamilton Depression Rating scale (HAMD). Furthermore, alterations in the metabolites 3-Indolepropionic acid, N-acetylphenylalanine, 3-Methylhistidine, Norepinephrine, Ortho-hydroxyphenylacetic acid, 3-Hydroxymethylglutaric acid and Indole-3-carboxaldehyde were associated with functional changes in key prefrontal regions, specifically, the Amplitude of Low-Frequency Fluctuations (ALFF) and Regional Homogeneity (ReHo) in the left superior frontal gyrus, and ReHo in the left middle frontal gyrus.

**Conclusions:**

This study uncovers preliminary evidence of a distinct metabolic signature in adolescent depression that normalizes following iTBS treatment and correlates with both prefrontal functional recovery and clinical improvement. The identified metabolites serve as potential candidate biomarkers and shed light on the brain-metabolism relationship. These findings offer preliminary candidate biomarkers for future investigations into interindividual variability in iTBS treatment response and offer a tentative framework for developing personalized neuromodulation strategies for adolescent depression.

**Clinical Trial Registration:**

The study has been registered on https://www.chictr.org.cn/ (registration number: ChiCTR2500106503 and ChiCTR2500113926; registration link: https://www.chictr.org.cn/showproj.html?proj=279390 and https://www.chictr.org.cn/showproj.html?proj=279455).

## Main Points

1. We uncovered a distinct panel of 17 plasma metabolites that are distinctly dysregulated in adolescent depression and undergo normalization subsequent to intermittent theta-burst stimulation (iTBS) treatment.

2. Changes in specific metabolites are closely correlated with clinical improvement, thereby establishing a direct link between metabolic recovery and the alleviation of depressive symptoms after iTBS.

3. Our findings provide support for a metabolite-brain function coupling framework: iTBS-induced modulation of specific prefrontal cortical activity (left superior frontal gyrus/left middle frontal gyrus [L-SFG/L-MFG]) is associated with the normalization of key metabolites linked to energy metabolism, neurotransmission, and gut-brain axis signaling.

## 1. Introduction

Major depressive disorder (MDD) in adolescents poses a substantial challenge to public health, carrying considerable economic and social consequences [1,2,3]. Globally, the prevalence of depressive symptoms in this age group has seen a notable increase [2]. In both China and the United States, the prevalence of depression among adolescents has been rising year on year [4,5]. Since its US Food and Drug Administration (FDA) approval in 2008, transcranial magnetic stimulation (TMS) has shown efficacy in treating depression [6,7], with its safety and effectiveness also confirmed in adolescent populations [8]. Accelerated intermittent theta-burst stimulation (iTBS), a form of TMS therapy characterized by durable efficacy and shorter treatment cycles [9], has gained particular popularity among adolescent depression patients and their families.

Extensive registry data indicate that while most patients benefit from TMS, approximately one-third of MDD patients do not respond to treatment [10,11]. This variability in treatment response among individuals may stem from abnormal brain activity in patients, particularly in emotion-processing neural circuits. Adolescents exhibit unique neurodevelopmental trajectories and structural vulnerability, characterized by the ongoing maturation of the prefrontal cortex—the region central to executive control and emotional regulation [12,13]. Critically, some evidence indicates that adolescent depression is associated with structural alterations in these very regions, including reduced cortical thickness in the Dorsolateral Prefrontal Cortex (DLPFC) and altered patterns of brain asymmetry [14,15]. Such morphometric changes may underlie the functional dysregulation observed in these circuits during emotional processing. To precisely capture brain dynamics during genuine emotional challenges faced by adolescents, our previous research employed functional magnetic resonance imaging within a naturalistic stimulus paradigm (N-fMRI) [16]. We discovered that under high-to-medium emotional arousal conditions, adolescent MDD patients exhibit abnormal activation in the left superior frontal gyrus (L-SFG) and left middle frontal gyrus (L-MFG). Consequently, the L-DLPFC/L-SFG represents not only a functional hotspot identified by our prior neuroimaging work but also a structurally susceptible node within the developing brain’s affect-regulation network. This confluence of functional and structural evidence solidifies the rationale for targeting this region with neuromodulation like iTBS, which may promote corrective neuroplastic changes.

To better understand the variability in treatment outcomes across individuals, this study integrated neuroimaging with metabolomic profiling. Resting-state functional magnetic resonance imaging (rs-fMRI) has been a cornerstone methodology in depression research, where derived metrics are frequently employed to examine distinctions between patients with depression and healthy individuals, as well as to track alterations pre- and post-intervention. Prior work has highlighted the relevance of Regional Homogeneity (ReHo) in characterizing MDD [17], whereas other investigations have emphasized the Amplitude of Low-Frequency Fluctuations (ALFF) as a pivotal measure for assessing depressed patients [18,19].

Moreover, as the ultimate product of interactions between gene expression, protein function, and the cellular environment [20], metabolomics demonstrates significant potential in identifying pathways of antidepressant response and the pathophysiological mechanisms underlying depression [21]. Research has already proposed that certain metabolites may serve as potential biomarkers [22,23]. Moreover, in recent years numerous studies have combined metabolomics techniques with TMS treatment for depression, suggesting that certain metabolites can significantly predict the efficacy of TMS therapy for depressive disorders [24,25]. Thus, whether metabolomics or magnetic resonance imaging data, both play a crucial role in TMS treatment for adolescent depression.

Therefore, this study integrates these methodologies, adopting a systematic perspective to investigate the issue of inter-individual variability in the efficacy of iTBS treatment for adolescents with depression. By integrating metabolomics, multi-modal neuroimaging, and clinical assessments, this study aims to reveal the relationship between brain and metabolism of iTBS treatment in adolescent depression, providing new perspectives for precision therapy.

We hypothesize that differential metabolites were identified in comparisons between adolescents with depression and healthy controls, as well as following iTBS treatment. These differential metabolites further correlated with brain functional metrics and clinical scales. Most importantly, we hypothesize that effective neuromodulation may operate through metabolite-brain function coupling—a bidirectional interaction where brain circuit activity regulates systemic metabolism, and metabolic shifts, in turn, influence neural function and clinical state. Metabolites may serve as potential biomarkers for therapeutic response to iTBS, with their underlying mechanisms linking to changes in brain function being preliminarily elucidated.

## 2. Materials and Methods

### 2.1 Participants

In this study, adolescent patients with depression were recruited from the outpatient department of Tianjin Anding Hospital. Prior to enrollment, all participants were fully informed of the study procedures and potential risks. Participants who met the inclusion and exclusion criteria were included in the study after providing written informed consent. Magnetic resonance imaging (MRI) data and blood samples for biomarker analysis were collected from all subjects both before and after iTBS treatment. All healthy participants were recruited via posters. They were also required to provide MRI data and blood samples, with data collected only once from each healthy participant.

The inclusion criteria were as follows: (1) aged 12–18 years; (2) diagnosed with depressive disorder by psychiatrists per Diagnostic and Statistical Manual of Mental Disorders, Fifth Edition (DSM-5) criteria and confirmed via Mini International Neuropsychiatric Interview for Children and Adolescents (MINI-KID); (3) Montgomery-Åsberg Depression Rating Scale (MADRS) score ≥14.

The exclusion criteria were as follows: (1) metallic implants above the chest (especially intracranial or cardiac devices); (2) severe neurological or major systemic illnesses (e.g., epilepsy, malignant tumors); (3) current psychotic symptoms; (4) substance/alcohol abuse history in the past year; (5) language/communication barriers affecting cooperation; (6) ineligibility deemed by clinicians/investigators.

The withdrawal/discontinuation criteria were as follows: (1) withdrawn informed consent; (2) intolerable adverse events or severe complications; (3) unqualified MRI and blood samples quality control.

### 2.2 Clinical Measurements

This study utilized the Hamilton Depression Rating scale (HAMD) and MADRS to assess patients’ levels of depression and overall disease severity. These assessments are conducted by professionally qualified measurement assessors who have undergone identical training.

### 2.3 Experimental Procedure

After providing informed consent, participants underwent baseline data collection, including psychological scale assessments (HAMD, MADRS), blood sample collection, and MRI scanning. Upon completion of the iTBS treatment course, patients underwent follow-up assessments, which included the same psychological scales, blood sample collection, and MRI scanning. For the patient group, data were collected at these two time points, whereas healthy controls completed a single data collection session.

### 2.4 Blood Sample Collection and Plasma Metabolomic Profiling

Blood samples were obtained by clinical staff following standardized protocols. Venous blood (3 mL) was collected into ethylenediaminetetraacetic acid (EDTA)-anticoagulant tubes and centrifuged at 4 °C and 3000 rpm for 13 minutes to isolate plasma. The plasma was then aliquoted into 300 µL aliquots and stored at –80 °C until further analysis. Metabolite profiling was carried out using ultra-high-performance liquid chromatography coupled with triple quadrupole mass spectrometry (Agilent 1290 Infinity LC, Agilent Technologies, Santa Clara, CA, USA). Following standardized quality control procedures, high-throughput targeted metabolite detection was performed. Subsequent data processing included the exclusion of metabolites with a relative standard deviation (RSD) exceeding 30% in quality-control samples, imputation of missing values via the k-nearest neighbors (KNN) algorithm, and removal of low-abundance metabolites detected in less than 50% of samples. Finally, data were processed with MetaboAnalyst 6.0 (https://www.metaboanalyst.ca/) [26], which involved log_10_ transformation and auto-scaling (mean-centering and unit variance scaling) to reduce technical variability.

### 2.5 Differential Metabolite Screening and Analysis

A metabolite was defined as significantly differential only when meeting all of the following criteria: False Discovery Rate (FDR) <0.05 after multiple comparisons correction, a variable importance in projection (VIP) score >1 derived from orthogonal partial least squares-discriminant analysis (OPLS-DA) analysis, and a fold change (FC >1.5 or <0.67). Initial screening relied on independent-samples *t*-tests, with* p*-values adjusted using the Benjamini-Hochberg method. Principal component analysis (PCA) was then applied to visualize group differences, and OPLS-DA was used to enhance group discrimination. Model validity was further assessed through permutation testing (1000 iterations). This analytical pipeline was employed both to identify baseline differences between patients and healthy controls and to detect metabolite changes following iTBS treatment within the patient group.

For biomarker screening, receiver operating characteristic (ROC) curve analysis was conducted to evaluate the capacity of each differential metabolite to distinguish adolescent depression patients from controls. Metabolites with an area under the curve (AUC) >0.8 were retained for presentation. Analysis was performed using MetaboAnalyst 6.0 software.

### 2.6 MRI Data Acquisition

Imaging data were acquired on a Siemens 3.0 T MRI scanner (MAGNETOM Prisma, Siemens, Munich, Germany) at Tianjin Anding Hospital. T1-weighted structural images were obtained with an MP-RAGE sequence using the following parameters: repetition time (TR) = 2000 ms, echo time (TE) = 2.32 ms, field of view (FOV) = 230 × 230 mm^2^, matrix size = 256 × 256, flip angle = 8°, slice thickness = 0.90 mm, number of slices = 208, voxel size = 0.9 × 0.9 × 0.9 mm^3^.

The rs-fMRI data were collected using a gradient-echo echo-planar imaging (GE-EPI) sequence with the following parameters: TR = 800 ms, TE = 30 ms, FOV = 208 × 208 mm^2^, matrix size = 104 × 104, flip angle = 56°, slice thickness = 2 mm, number of slices = 72, voxel size = 2 × 2 × 2 mm^3^, and 450 volumes acquired.

For the naturalistic stimulation task [16], participants were asked to watch silent video clips while maintaining head stability. fMRI data during this task were also acquired with a GE-EPI sequence using identical parameters (TR = 800 ms, TE = 30 ms, FOV = 208 × 208 mm^2^, matrix size = 104 × 104, flip angle = 56°, slice thickness = 2 mm, number of slices = 72, voxel size = 2 × 2 × 2 mm^3^), with a total of 614 volumes collected.

### 2.7 MRI Data Analysis

#### 2.7.1 Data Preprocessing

Data preprocessing was carried out using DPARSF software [27] (V5.3_210101, http://rfmri.org/DPARSF), adhering to standard pipeline provided by the toolbox, within the MATLAB R2020a environment (Mathworks, Inc., Sherborn, MA, USA).

For rs-fMRI data, the initial 10 volumes of each scan were removed to allow for magnetic field equilibration. Head motion correction was performed via realignment. T1-weighted structural images were co-registered to the functional images and subsequently processed with segmentation and normalization using the Diffeomorphic Anatomical Registration using Exponentiated Lie algebra (DARTEL) approach. Signals from white matter (WM) and cerebrospinal fluid (CSF), global signal, head motion parameters (based on Friston’s 24-parameter model), and polynomial trends were regressed out as nuisance covariates. Finally, a temporal band-pass filter (0.01–0.1 Hz) was applied.

Preprocessing of naturalistic fMRI data was conducted using SPM12 (Wellcome Centre for Human Neuroimaging, London, UK). The final 27 volumes of each scan were discarded, leaving 587 volumes for further analysis. Steps included motion correction, spatial realignment, and co-registration of the high-resolution T1 image with the mean functional volume from realignment. Subsequent segmentation and normalization using DARTEL produced images with a 2 × 2 × 2 mm^3^ voxel size. Nuisance regression was performed to remove WM and CSF signals, global signal, head motion parameters, and polynomial trends. Functional images were then normalized into Montreal Neurological Institute (MNI) space and spatially smoothed with a Gaussian kernel (full width at half maximum, FWHM = 6 × 6 × 6 mm^3^).

#### 2.7.2 Data Analysis

Following preprocessing, ALFF analysis was applied to the resting-state data [28]. Data were first smoothed with a 6 mm FWHM Gaussian kernel. The time series was then transformed into the frequency domain via Fast Fourier Transform to obtain the power spectrum. The square root of the power within the 0.01–0.1 Hz range was computed for each voxel. ALFF values were then standardized by dividing each voxel’s ALFF by the global mean ALFF, generating normalized whole-brain ALFF maps.

ReHo (expressed as Kendall’s coefficient of concordance, KCC) calculation, the spatially normalized data were band-pass filtered (0.01–0.1 Hz) to minimize low-frequency drift and high-frequency noise [29]. ReHo was derived by assessing the temporal similarity between a given voxel and its 27 nearest neighbors. The resulting ReHo maps were smoothed with a 6 mm FWHM Gaussian kernel to reduce residual noise.

Analysis of naturalistic stimulation task data employed a general linear model (GLM) to examine fMRI signals acquired during video viewing. After first-level contrasts comparing adolescent MDD patients with healthy controls, spherical regions of interest (ROIs, 6 mm radius) were centered on the two peak coordinates identified from whole-brain analysis [30]. Beta values were extracted from these ROIs for each participant pre- and post-treatment. Treatment-related changes in neural activity were represented by the difference in Beta values (post-treatment minus pre-treatment).

Finally, ALFF and ReHo values from the resting-state data were also extracted specifically at the two peak coordinates derived from the naturalistic stimulation analysis, providing corresponding functional measures at these locations.

### 2.8 iTBS Treatment

Following the initial cranial MRI scan, the targeting coordinates were determined by researchers specialized in data analysis and subsequently transmitted to the iTBS treatment team. A subset of patients received stimulation targeting the coordinate (MNI: –41, 16, 54) [31] in standard space. Following completion of iTBS treatment, the therapist documented the patient’s condition during the session and inquire whether any adverse reactions have occurred.

iTBS treatment comprises two specific protocols: a two-day or four-day accelerated course. The two-day accelerated treatment is completed consecutively, whereas the four-day accelerated treatment requires a 5–7 day rest period after the second day’s session before completing the remaining two days of iTBS. Each treatment session lasts 10 minutes, with 50-minute intervals between sessions. Five cycles per day were administered, totaling 20 sessions of iTBS, a high-frequency TMS protocol with brief stimulation bursts. Specific stimulation parameters comprised 100% Resting Motor Threshold (RMT), 50 Hz intra-burst frequency, 5 Hz inter-burst frequency, 2-second stimulation duration, and 8-second inter-stimulus intervals, with each treatment delivering 1800 pulses. The accelerated treatment protocol over two days delivered a total of 18,000 pulses, while the four-day accelerated treatment protocol delivered a total of 36,000 pulses.

### 2.9 Statistical Analysis

In the descriptive statistical analysis, the normality of continuous variables was assessed using the Shapiro–Wilk test. Variables conforming to a normal distribution (*p* > 0.050) were reported as mean ± standard deviation (SD), while those with a skewed distribution were expressed as median (interquartile range, IQR). Categorical variables were presented as counts (percentages). Subsequently, intergroup comparisons were conducted: continuous variables were compared using the independent-samples *t*-test for normally distributed data or the Mann-Whitney U test for skewed data, and categorical variables were analyzed via the Chi-square test. Paired-sample *t*-tests were conducted on pre- and post-treatment scores for 52 participants with MADRS data and 42 participants with HAMD-17 data, excluding those with partially missing questionnaires.

Subsequently, the change in differential metabolites (those that differed between the healthy control and patient groups at baseline and also changed post-treatment) was calculated as the difference between pre- and post-treatment levels. First, Pearson’s correlation analysis was performed to evaluate the linear relationships between changes in differential metabolites and changes in clinical efficacy measures, including MADRS and HAMD reduction scores, as well as neuroimaging parameters. Next, multivariate linear regression models were constructed to further investigate the independent and quantitative associations between metabolite changes and the aforementioned clinical and neuroimaging outcomes, with adjustment for potential confounding factors where appropriate. After excluding participants with partial missing data, the final neuroimaging dataset included changes in resting-state ALFF and ReHo values from 47 participants, and changes in beta values derived from the naturalistic stimulation task in 28 participants.

To explore the relationship between differential metabolites and the treatment target, we also extracted pre- to post-treatment changes in the fixed target region (MNI: –41, 16, 54) for the 32 patients who received stimulation at this coordinate and analyzed their relationship with changes in the differential metabolites. All statistical analyses were performed using SPSS 26.0 software (IBM Corp, Armonk, NY, USA), with statistical significance set at *p *< 0.050 (two-tailed).

## 3. Results

### 3.1 Demographic Information

The majority of the patients completed the full course of the accelerated iTBS treatment protocol. For the core metabolomic analysis, which required qualified paired plasma samples collected both before and after treatment, complete data were available for 53 patients. The remaining participants were excluded from this analysis due either to refusal of blood collection at one of the required time points or to failure of the samples to meet quality control standards. Following completion of iTBS treatment, the vast majority of adolescents reported no adverse events or tolerability issues. A small number of subjects experienced transient symptoms during the initial treatment session, including mild manifestations such as dizziness and nausea.

The demographic characteristics of the adolescent depression patients and healthy controls are presented in Table 1. The results indicated no significant differences in gender, age or body mass index (BMI) between the two groups. Regarding clinical scales, both HAMD and MADRS scores demonstrated significant changes. The change in HAMD score following treatment is 6.62 ± 4.32. The change in MADRS score following treatment is 9.30 ± 6.54. Specifically, paired-sample *t*-tests revealed significant differences in HAMD-17 (t_(41)_ = 9.94, *p* < 0.001) and MADRS (t_(51)_ = 10.27, *p* < 0.001) scores, indicating symptomatic relief after iTBS treatment. For detailed information, please refer to **Supplementary Table 1**.

**Table 1.** **Demographic characteristics of the participants**.

Items	MDD	HC	Statistical analysis	
			Z/t/χ^2^	*p*
Sample size	85	48	–	–
Sex (M/F)	22/63	19/29	2.700	0.100
Age (year)	15.00 (14.00, 16.50)	16.00 (12.25, 18.00)	–1.161	0.246
BMI	20.00 (17.35, 23.44)	21.37 (18.37, 24.64)	–1.333	0.183
HAMD-17	20.64 ± 5.53	–	–	–
MADRS	29.38 ± 6.60	–	–	–

M, male; F, female; BMI, body mass index; HC, healthy control subjects; MDD, major depressive disorder; HAMD, Hamilton Depression Rating scale; MADRS, Montgomery-Åsberg Depression Rating Scale.

### 3.2 Differential Metabolites

**Fig. 1.**
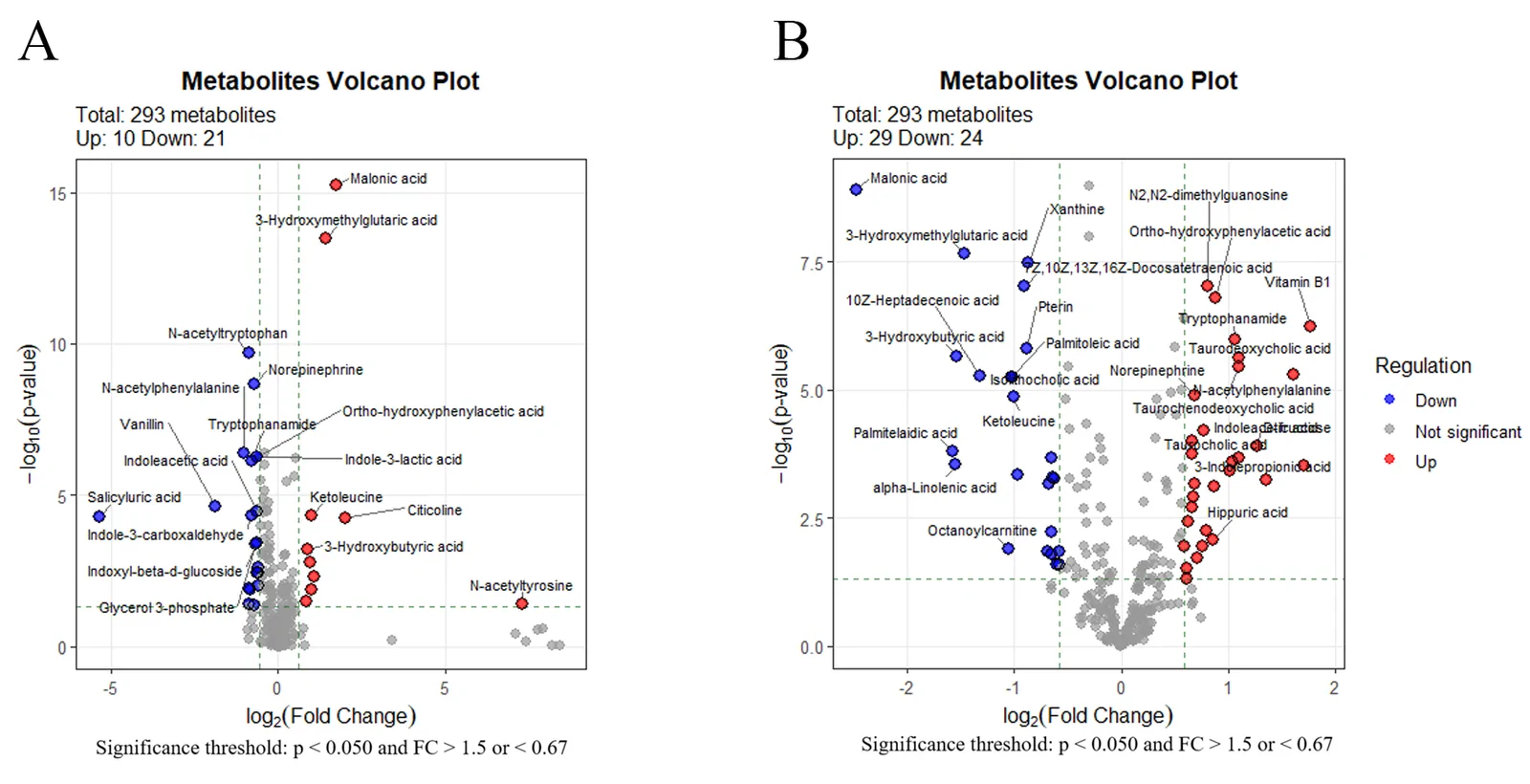
**Volcano plot of metabolites**. A total of 293 metabolites were analyzed. (A) Comparison between the patient group and healthy controls. A total of 31 differentially expressed metabolites were identified—10 upregulated and 21 downregulated. (B) Comparison between pre-treatment and post-treatment in the patient group. A total of 53 differentially expressed metabolites were identified—29 upregulated and 24 downregulated. FC, fold change.

A total of 293 metabolites were initially analyzed. As shown in Fig. 1A, when comparing the patient group with the healthy control group before treatment and applying screening criteria, 31 metabolites were identified—10 upregulated and 21 downregulated. After further screening with OPLS-DA analysis, 30 differential metabolites remained.

As shown in Fig. 1B, for the post-treatment comparison within the patient group, the same 293 metabolites were screened using the same criteria, identifying 53 metabolites—29 upregulated and 24 downregulated. After applying the OPLS-DA analysis, 46 differential metabolites remained.

The final set of differential metabolites was obtained by taking the intersection of these two sets—i.e., metabolites that differed between patients and controls at baseline and also changed after iTBS treatment. We subsequently took the intersection of these two sets of differential metabolites and identified 17 metabolites that remained significant. This indicates that these 17 metabolites not only differed between adolescent MDD patients and healthy controls but also showed significant alterations following iTBS treatment. Detailed data for these differential metabolites are available in the **Supplementary Tables 2 and 3**.

**Fig. 2.**
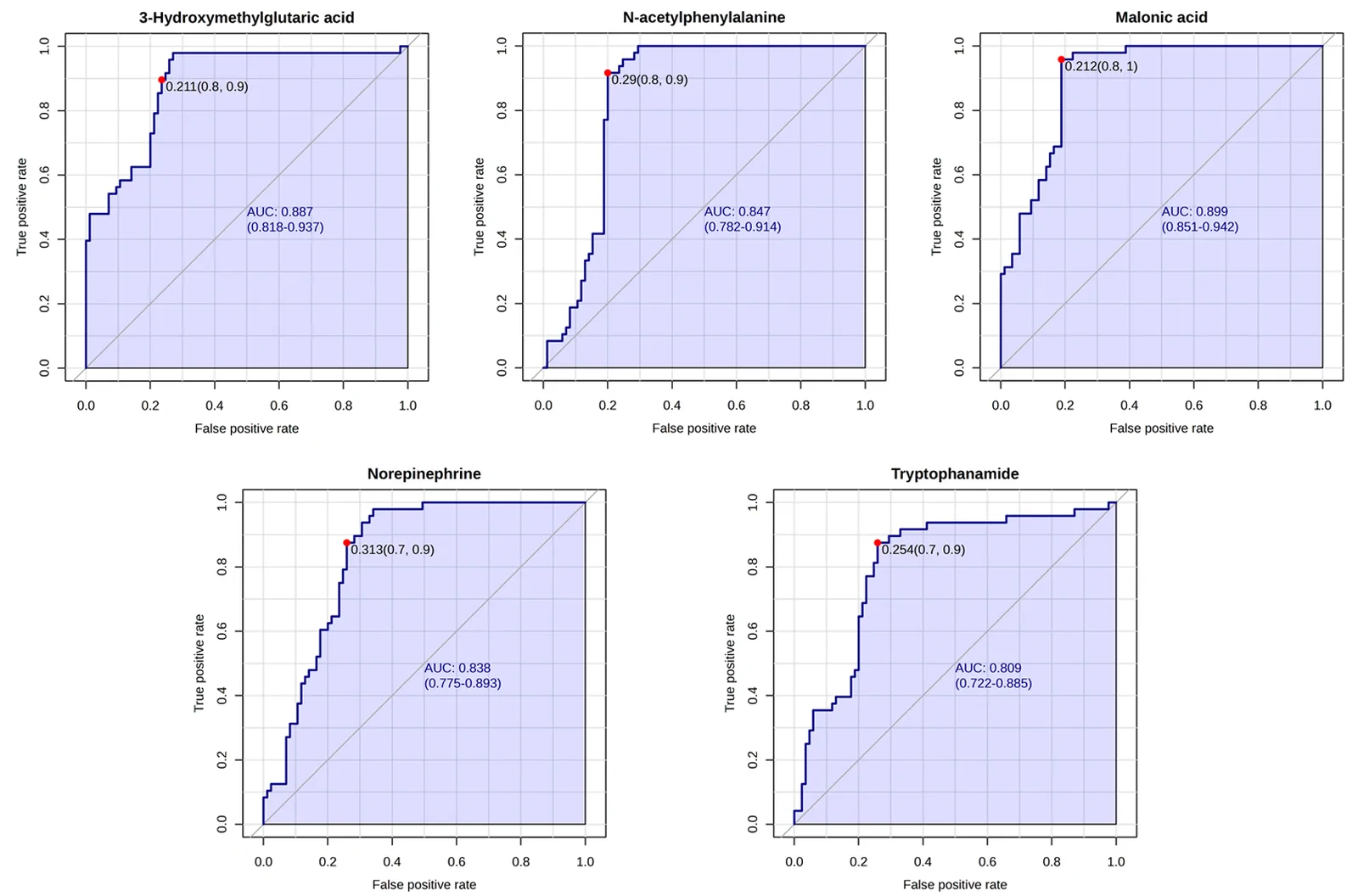
**Receiver operating characteristic curves for metabolites differing partially between adolescents with depression and healthy controls**. Receiver operating characteristic (ROC) curves for the top 5 differential metabolites, including corresponding optimal cutoff values (thresholds) determined by the point closest to the top-left corner of the curve. Each ROC curve reflects a specific metabolite’s ability to distinguish adolescents with depression from healthy controls, with the area under the curve (AUC) serving as a measure of diagnostic accuracy. The optimal cutoff point for each metabolite, which balances sensitivity and specificity, is indicated on the curve. The dotted diagonal line represents the performance of a random classifier (AUC = 0.5), while higher AUC values indicate better diagnostic potential for each metabolite.

We further performed ROC curve analysis on these 17 differential metabolites from the adolescent MDD patients versus healthy controls comparison. Ten metabolites showed an AUC >0.7. As shown in Fig. 2, we present five differential metabolites with AUC values exceeding 0.8, including 3-Hydroxymethylglutaric acid, Malonic acid, N-acetylphenylalanine, Norepinephrine and Tryptophanamide, which demonstrate a robust capacity to effectively distinguish adolescents with depression from healthy controls.

### 3.3 Regression Analysis of Differential Metabolites, Neuroimaging Data, and Clinical Scales

**Fig. 3.**
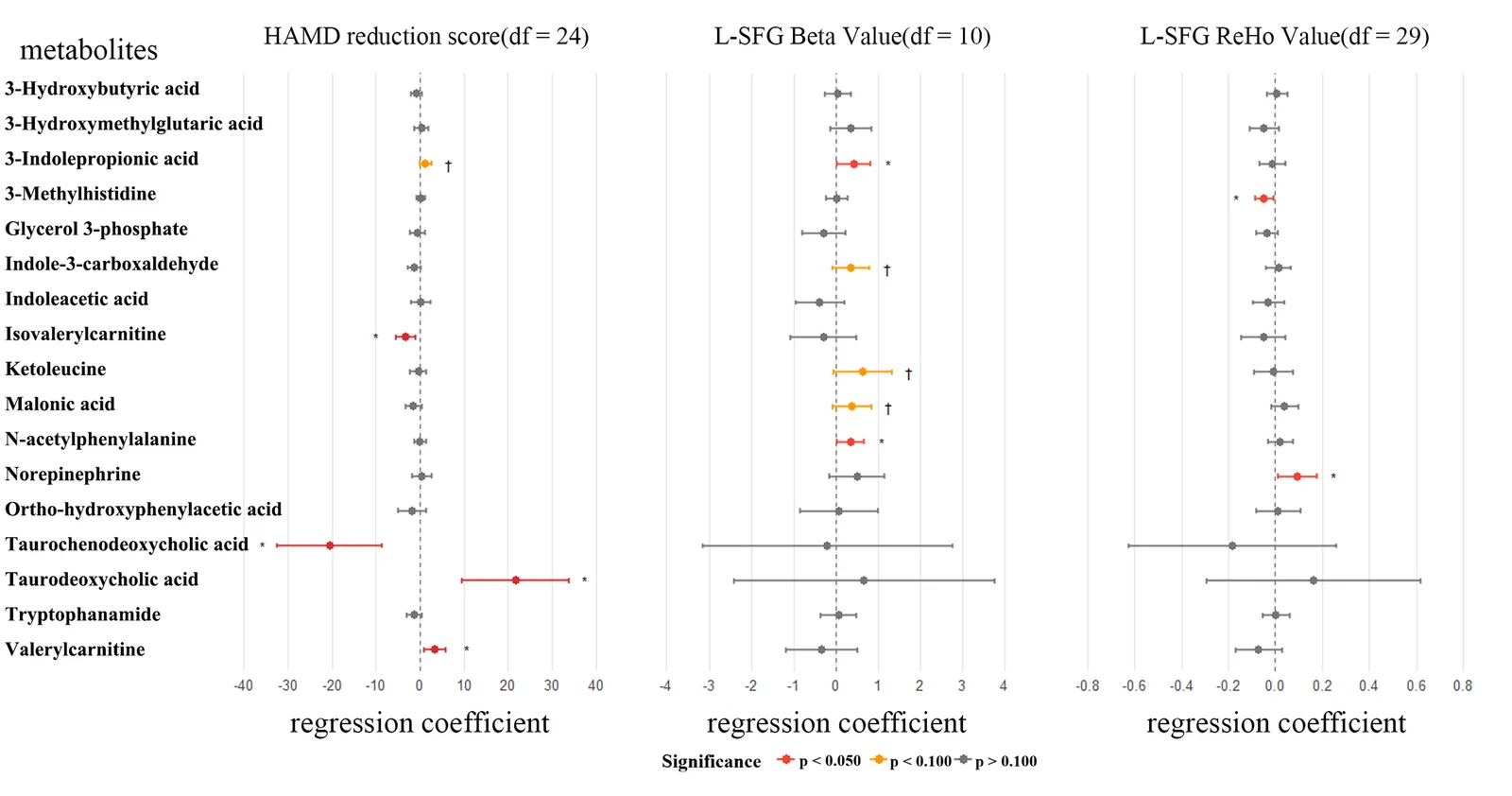
**Regression forest plot of differential metabolites with clinical scales and imaging data**. The names of the 17 differentially abundant metabolites identified through screening are plotted along the vertical axis, while the regression coefficient values of the metabolites are plotted along the horizontal axis. Each point in the figure represents the regression coefficient of a specific metabolite, and each horizontal line represents the 95% confidence interval for that regression coefficient. Points are color-coded as follows: red and asterisk (*) indicates a significance level of *p* < 0.050, orange and dagger (†) indicates* p* < 0.100, and grey indicates *p* ≥ 0.100. The degrees of freedom (df) reported are for the residuals. L-SFG, left superior frontal gyrus.

As shown in Fig. 3, the left panel presents the results of an exploratory regression analysis between the HAMD reduction scores and the change values of the metabolites. The overall model relating HAMD reduction scores to changes in metabolites was significant (*p* = 0.049) and explained 60% of the variance (R^2^ = 0.60). Among the 17 differential metabolites, four metabolites (Isovalerylcarnitine, Taurochenodeoxycholic acid, Taurodeoxycholic acid, and Valerylcarnitine) showed statistically significant associations (*p* = 0.007, 0.002, 0.001, 0.009), and one metabolite (3-Indolepropionic acid) showed a non-significant trend (*p* = 0.064). Notably, the AUC values for these metabolites were 0.68, 0.61, 0.61, 0.68, and 0.66, respectively.

The middle section shows the results of an exploratory regression analysis between the change values of the N-fMRI data at the L-SFG location and the change values of the metabolites. The model for L-SFG beta value changes was not statistically significant (*p* = 0.176), despite a high explained variance (R^2^ = 0.752). Two metabolites (3-Indolepropionic acid and N-acetylphenylalanine) demonstrated significant associations (*p *= 0.033, 0.039), while three metabolites (Indole-3-carboxaldehyde, Ketoleucine, and Malonic acid) exhibited non-significant trends (*p* = 0.091, 0.066, 0.086).

The right section presents the exploratory regression analysis between the change values of the ReHo measure from the rs-fMRI data at the L-SFG location and the change values of the metabolites, which revealed that two metabolites (3-Methylhistidine and Norepinephrine) showed significant associations (*p* = 0.029, 0.027). The model for L-SFG ReHo changes was significant (*p* = 0.025) with an R^2^ of 0.570.

### 3.4 Correlation Results Between Neuroimaging Data and Differential Metabolites

**Fig. 4.**
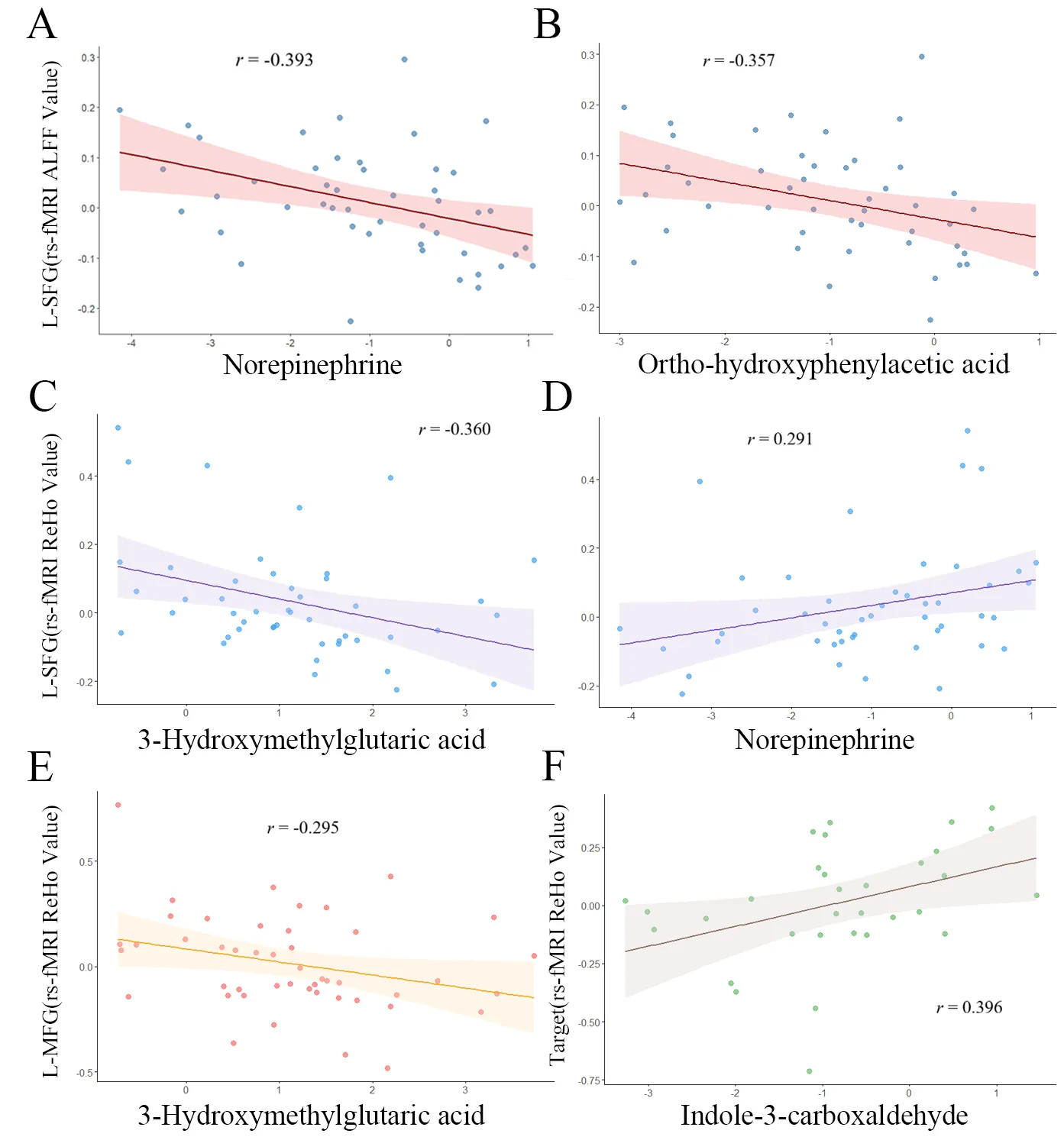
**Scatter plot correlating differentially expressed metabolites with imaging data**. The horizontal axis represents the change values of differentially expressed metabolites, while the vertical axis represents the change values of imaging data. All correlations were significant at *p* < 0.050, r denotes the correlation coefficient. (A,B) Correlation between resting-state ALFF variation at L-SFG locations and differential metabolites Norepinephrine (A) and Ortho-hydroxyphenylacetic acid (B). (C,D) Correlation between resting-state ReHo variation at L-SFG locations and differential metabolites 3-Hydroxymethylglutaric acid (C) and Norepinephrine (D). (E) Correlation between resting-state ReHo variation at L-MFG locations and differential metabolites 3-Hydroxymethylglutaric acid. (F) Correlation between resting-state ReHo changes at therapeutic target locations (MNI: –41, 16, 54) and differential metabolites Indole-3-carboxaldehyde. ALFF, Amplitude of Low-Frequency Fluctuations; L-SFG, left superior frontal gyrus. ReHo, Regional Homogeneity; L-MFG, left middle frontal gyrus; MNI, Montreal Neurological Institute.

In the correlation analysis conducted between the change values in neuroimaging data and the differential metabolites. As shown in Fig. 4A,B, in the results from the rs-fMRI data, we found significant results in the correlation analysis between ALFF difference values in the L-SFG and differential metabolites (Norepinephrine, Ortho-hydroxyphenylacetic acid) (r = –0.393, *p* = 0.006; r = –0.357, *p* = 0.014). That is, the differential metabolites Norepinephrine and Ortho-hydroxyphenylacetic acid showed significant negative correlations with ALFF difference values in the L-SFG.

As shown in Fig. 4C,D, significant results were also found in the correlation analysis between ReHo difference values at the same location and differential metabolites (3-Hydroxymethylglutaric acid, Norepinephrine). Specifically, the differential metabolite 3-Hydroxymethylglutaric acid showed a significant negative correlation with ReHo values in the L-SFG (r = –0.360, *p* = 0.013); the differential metabolite Norepinephrine showed a significant positive correlation with ReHo difference values in the L-SFG (r = 0.291, *p* = 0.048).

Similarly, as shown in Fig. 4E, significant results were found in the correlation analysis between ReHo difference values in the L-MFG and the differential metabolite (3-Hydroxymethylglutaric acid). Specifically, the differential metabolite 3-Hydroxymethylglutaric acid showed a significant negative correlation with ReHo difference values in the L-MFG (r = –0.295, *p* = 0.044).

In the subset of participants receiving fixed-target treatment. As shown in Fig. 4F, analysis of the ReHo difference values and differential metabolites in rs-fMRI at fixed target locations revealed significant results for certain metabolites. Specifically, the differential metabolite Indole-3-carboxaldehyde showed a significant positive correlation with ReHo difference values under rs-fMRI at the treatment fixed target site (r = 0.396, *p* = 0.025).

## 4. Discussion

This study, through the integration of plasma metabolomics and multimodal brain imaging data, identified 17 key differential metabolites in adolescent patients with depression. These metabolites not only showed significant differences from healthy controls but also underwent significant changes after iTBS treatment. The area under the curve for metabolites 3-hydroxymethylglutaric acid, malonic acid, N-acetylphenylalanine, norepinephrine and tryptophanamide exceeds 0.8. The metabolites isovalerylcarnitine, taurochenodeoxycholic acid, taurodeoxycholic acid, and valerylcarnitine were significantly correlated with the percentage reduction in scores on the HAMD. It is noteworthy that these metabolites possess predictive value, particularly Valerylcarnitine and Isovalerylcarnitine, which exhibit the highest AUC values and may represent promising candidate indicators of treatment response—though their utility as formal predictive biomarkers requires further validation. The metabolites 3-Indolepropionic acid, N-acetylphenylalanine, 3-Methylhistidine, Norepinephrine, Ortho-hydroxyphenylacetic acid, 3-Hydroxymethylglutaric acid and Indole-3-carboxaldehyde alterations were correlated with functional indicators in specific brain regions (L-SFG and L-MFG). This study provides preliminary evidence for the relationship between brain and metabolism underlying iTBS treatment for adolescent depression, offering potential candidate metabolic markers and fresh therapeutic perspectives for iTBS therapy—with clinical applicability contingent on independent replication.

Research on these 17 metabolites enables their broad classification into several categories. The first category is related to energy metabolism and mitochondrial function, including 3-Hydroxybutyric acid, Glycerol 3-phosphate, Malonic acid, Ketoleucine, Isovalerylcarnitine and Valerylcarnitine. Some studies have found differences in 3-Hydroxybutyric acid [32] between depression patients and healthy controls. This change may reflect abnormalities in energy metabolism associated with adolescent depression. For instance, a recent large-scale study of over 2500 adults confirmed that reduced acetylcarnitine (C2) levels constitute a stable feature of depression, while elevated propionylcarnitine (C3) correlates with depressive severity. Both alterations are specifically associated with “atypical/energy-related” symptoms such as fatigue and hypersomnia [33,34,35]. Our study not only validated the central role of acylcarnitines in an adolescent cohort distinct from adult populations, but also refines the characterization of metabolic disruption—shifting from the broad “short-chain acylcarnitine” category to more specific biological pathways by identifying Isovalerylcarnitine (a branched-chain amino acid metabolism marker) and Valerylcarnitine (a fatty acid oxidation marker).

The second category includes neurotransmitters and neuromodulation-related substances, such as Norepinephrine, Ortho-hydroxyphenylacetic acid and N-acetylphenylalanine. In particular, Norepinephrine, as both a monoamine neurotransmitter and a hormone, has been extensively demonstrated through research to be closely associated with depression [22,36,37]. Wang et al.’s study [22] clearly shows that the neurotransmitter metabolic patterns of first-episode, untreated adolescent depression patients differ fundamentally from those of adult patients: adults exhibit widespread neurotransmitter system dysregulation, particularly comprehensive deficits in the tryptophan metabolic pathway; whereas adolescent patients’ disturbances are more concentrated within the catecholamine system. We observed significant alterations in norepinephrine levels, which align closely with the aforementioned research emphasizing catecholaminergic system dysfunction in adolescent depression. As a key stress and arousal neurotransmitter, imbalances in norepinephrine may be closely associated with symptoms commonly observed in adolescents with depression.

The third category consists of metabolites related to the gut-microbiome-brain axis, such as Indoleacetic acid, 3-Indolepropionic acid and Indole-3-carboxaldehyde. Previous research [38] has found that gut microbiota-derived Indole-3-carboxaldehyde (I3C) in depression patients can act as a gut-brain signaling molecule, linking gut microbial tryptophan metabolism to the host’s stress vulnerability. The gut microbiome-derived-I3C can help buffer the host’s stress through the aryl hydrocarbon receptors (AhR)/SOCS2/NF-κB/NLRP3 pathway, providing a gut-brain signaling basis for emotional behavior. Apart from these three categories, other metabolites, such as Taurodeoxycholic acid is a confounding factor in MDD, and its interaction with gut microbiota may be a pathophysiological mechanism of MDD [39].

After analyzing the changes in these metabolites alongside clinical scales, we found metabolite changes synchronize with improvements in clinical symptoms. In the analysis with MRI imaging data, we found significant results in the regression analysis of natural stimulation imaging data for the L-SFG location with the differential metabolites. These findings suggest a possible metabolite-brain function coupling relationship. Central to our interpretation is the concept of metabolite-brain function coupling, which we define as a bidirectional, physiologically integrated relationship wherein activity in specific neural circuits modulates peripheral metabolic pathways, and shifts in these metabolic pathways reciprocally influence neural synchrony, network efficiency, and ultimately, behavior and clinical symptoms. Research indicates that modulating the concentrations of specific metabolites and inflammatory markers may alter functional connectivity within patients’ neural networks [40,41]. Similarly, by stimulating abnormal brain regions, we may also modify the levels of particular metabolites, thereby enabling their use as therapeutic biomarkers.

Thus, our findings support a preliminary metabolite-brain function coupling model underlying iTBS efficacy in adolescent depression. We propose that iTBS, by targeting the L-DLPFC/SFG, directly modulates aberrant neural activity at both the local and network levels. This neural modulation, in turn, may regulate systemic or local metabolic pathways—particularly those involving mitochondrial energetics, neurotransmitter systems, and gut-brain axis metabolites—as reflected in our identified plasma metabolite panel. The subsequent normalization of these metabolic profiles is associated with improvements in prefrontal functional metrics and, crucially, with reductions in clinical symptoms. This model invites a broader conceptual framework: iTBS can be regarded as a focal, high-intensity “dose” of targeted neuromodulation. The recovery induced by such targeted neuromodulation may involve mechanisms that extend beyond alterations in local neural activity. Recent studies on recovery following brain injury have demonstrated that effective functional recovery is often accompanied by dynamic interhemispheric interactions and the reorganization of distributed neural networks [42]. The effects of iTBS may converge, at both metabolic and functional levels, with those of other chronic, more diffuse “doses” of salutary environmental inputs. For instance, supportive peer interactions and regular physical activity—both well-documented to shape prefrontal structure/function and resilience in adolescence [43]—may also engage similar neuroplastic and metabolic pathways. Thus, the metabolic signatures we identified could represent part of a shared biological substrate through which diverse external factors—technological or psychosocial—exert their protective effects.

This study also has certain limitations. First, due to the integration of multiple metrics, the sample sizes varied across different indicators. Future experiments should be designed with greater rigour, with consistency maintained in the number of participants across different outcome metrics. Second, this study primarily establishes correlations rather than causality, so subsequent research should employ more rigorous experimental designs to investigate causal relationships. Additionally, without a longitudinal control group or a sham-stimulation arm, it is difficult to rule out the effects of time or placebo on the observed metabolic and neural changes. Future experiments should incorporate control groups to exclude temporal effects or placebo effects. Furthermore, to address potential test-retest variability in metabolite detection, repeat testing of healthy controls should be implemented, and rigorous procedures should be implemented in our metabolomic workflow. Finally, the retrospective registry used in this study deviates from international best practice regarding prospective clinical trial registries, and substantial modifications to the iTBS treatment protocol may also have introduced residual cohort heterogeneity.

## 5. Conclusions

In summary, this study systematically revealed distinct metabolite profiles in adolescent depression patients that correlate closely with prefrontal function and clinical symptoms by integrating plasma metabolomics with multimodal brain functional imaging techniques. These metabolites not only exhibit significant differences between patients and healthy controls but also demonstrate a marked reversal trend following iTBS treatment, showing high correlation with neural activity changes in specific brain regions (L-SFG and L-MFG). From a metabolite-brain function coupling perspective, this study provides initial insights into the potential mechanisms underlying iTBS treatment for adolescent depression. It offers preliminary candidate biomarkers for future investigations into treatment response variability among individuals undergoing iTBS therapy and establishes a tentative framework for developing personalized neuromodulation strategies for adolescent depression—with the caveat that these findings are exploratory and require independent replication and further validation before clinical or biomarker implications can be fully realized.

## Data Availability

The raw fMRI data and clinical scale data supporting the findings of this study are not publicly available due to privacy and ethical restrictions concerning the adolescent participant population. The data were collected under the approval of the Ethics Committee of Tianjin Anding Hospital and are subject to confidentiality agreements. However, the data are available from the corresponding author (Bin Zhang, zhang.bin845@foxmail.com) upon reasonable request and with permission from the institutional ethics committee.
