# Supplementary material for: Metabolic-Brain Functional Coupling Mechanism Underlying iTBS Treatment for Adolescent Depression
Source: Alpha Psychiatry. 2026 Aug 6;27(4):48591. doi: 10.31083/AP48591 (PMC13539994; doi:10.31083/AP48591)
Supplement: Supplementary file 1 [file 2757-8038-27-4-48591-s1.zip › Supplemental Material.docx]

**Supplemental materials**

1. **reliability coefficient of the scale**

The original validation studies reported high internal consistency for these scales (e.g., Cronbach’s α > 0.80). In the present study, we assessed scale reliability using our sample data. The internal consistency (Cronbach’s α) at baseline was 0.640 for HAMD-17 and 0.634 for MADRS; at post-treatment, it was 0.806 for HAMD-17 and 0.791 for MADRS.

2. **Detailed Description and Validation of the Naturalistic fMRI (N-fMRI) Paradigm**

**2.1. Source and Rationale of Video Stimuli**

The naturalistic video stimuli used in this study were adopted from a previously published and validated paradigm developed by Zhang et al (2022). This paradigm was specifically designed to elicit robust and ecologically valid emotional and social responses in adolescent populations. We selected this paradigm due to its proven efficacy in activating circuits involved in socio-emotional processing—a core domain impaired in adolescent depression.

**2.2. Content Description and Emotional Salience**

The video compilation has a total duration of 7 minutes and 50 seconds. It was edited from four public service announcement (PSA) clips and cartoon segments. The content was strategically chosen to depict scenarios highly relevant to adolescent experiences, primarily focusing on:

School-related social stress: Scenes involving peer interaction, performance evaluation, and social evaluation.

Emotional family interactions: Scenes depicting conflicts, reconciliations, and communications between children and parents.

To eliminate potential confounds from linguistic processing and ensure cross-cultural consistency, the audio track and all subtitles were removed (Honey et al., 2012). Participants watched the silent video, directing their attention purely to the visual social and emotional narratives.

**2.3. Stimulus Validation and Arousal Quantification**

Prior to its use in the main study, the video material underwent rigorous validation to quantify its emotional impact:

Raters: 33 independent raters (without emotional disorders) watched the video.

Segmentation: The full video was divided into 59 segments, each lasting 8 seconds (the final segment was 6 seconds).

Rating Task: For each segment, raters provided continuous ratings on the dimension of emotional arousal (i.e., the intensity of emotional activation).

Normative Arousal Levels: Based on the aggregate ratings, each segment was assigned a normative arousal value. Segments were then categorized into three discrete levels: Low, Medium, and High Arousal.

Stimulus Selection for Analysis: To create clear experimental conditions, segments representing the top, middle, and bottom 15% of the arousal distribution were selected for each level, resulting in 9 segments per arousal level (27 segments total). This allowed us to model brain activity in response to High vs. Medium Arousal contrasts, which formed the basis of our primary analysis.

2.4. **The Basis for Task Paradigm Activation of Relevant Neural Circuits**

By analyzing imaging data from natural stimulus videos using generalized linear models (GLM), we identified significant clusters demonstrating inter-group differences at the group level analysis of natural stimulus data, following cluster-level FWE correction. Specifically, at the High vs Medium level of the emotional arousal dimension, corrected points were observed in the left superior frontal gyrus (L-SFG) and middle frontal gyrus (L-MFG).

**Reference**

Zhang, Q., et al., *Comparing the effectiveness of brain structural imaging, resting-state fMRI, and naturalistic fMRI in recognizing social anxiety disorder in children and adolescents.* Psychiatry Research: Neuroimaging, 2022. **323**: p. 111485.

Honey, C.J., et al., Not lost in translation: neural responses shared across languages. Journal of Neuroscience, 2012. 32(44): p. 15277-15283.

**Supplementary Table 1 Demographic characteristics of the participants.**

| Items | MDD | | Statistical analysis | |
| --- | --- | --- | --- | --- |
|  | before | after | *t* | *p* |
| Sample size | 85 | 53 | _ | _ |
| Sex (M/F) | 22/63 | 14/39 | _ | _ |
| HAMD-17 | 20.64 ± 5.53 | 14.02 ± 6.98 | 9.94 | < 0.001 |
| MADRS | 29.38 ± 6.60 | 20.08 ± 8.40 | 10.27 | < 0.001 |

M, male; F, female; MDD, patients with major depressive disorder at baseline; HAMD, Hamilton Depression scale; MADRS, Montgomery-Åsberg Depression Rating Scale.

**Supplementary Table 2. Thirty differentially expressed metabolites identified in a comparison between adolescent depression patients and healthy controls.**

| **Metabolite Name** | ***t*** | ***p*-value** | **FDR** | **FC** | **VIP** |
| --- | --- | --- | --- | --- | --- |
| 3-Hydroxybutyric acid | 4.078 | 7.830E-05 | 6.198E-04 | 1.813 | 2.368 |
| 3-Hydroxymethylglutaric acid | 9.404 | 2.290E-16 | 3.360E-14 | 2.661 | 3.331 |
| 3-Indolepropionic acid | -2.978 | 3.460E-03 | 1.353E-02 | 0.565 | 2.028 |
| 3-Methylhistidine | -3.127 | 2.176E-03 | 1.009E-02 | 0.656 | 1.716 |
| 9-Hydroxy-10E,12Z-octadecadienoic acid | -3.050 | 2.771E-03 | 1.177E-02 | 0.553 | 1.996 |
| Citicoline | 4.737 | 5.570E-06 | 5.830E-05 | 3.995 | 2.065 |
| Cystathionine | -2.501 | 1.360E-02 | 4.241E-02 | 0.615 | 1.341 |
| Glycerol 3-phosphate | -4.211 | 4.700E-05 | 4.050E-04 | 0.629 | 1.866 |
| Indole-3-carboxaldehyde | -4.813 | 4.040E-06 | 4.740E-05 | 0.569 | 2.214 |
| Indole-3-lactic acid | -6.044 | 1.460E-08 | 5.360E-07 | 0.650 | 2.015 |
| Indoleacetic acid | -4.907 | 2.700E-06 | 3.590E-05 | 0.634 | 2.011 |
| Indoxyl-beta-d-glucoside | -4.227 | 4.420E-05 | 3.923E-04 | 0.635 | 1.711 |
| Isovalerylcarnitine | -3.473 | 6.981E-04 | 3.819E-03 | 0.634 | 1.676 |
| Ketoleucine | 4.803 | 4.200E-06 | 4.740E-05 | 1.977 | 2.013 |
| Malonic acid | 10.240 | 1.940E-18 | 5.680E-16 | 3.301 | 3.913 |
| Maltotriose | 3.359 | 1.026E-03 | 5.183E-03 | 2.098 | 1.499 |
| N-acetylphenylalanine | -6.158 | 8.410E-09 | 4.210E-07 | 0.487 | 2.977 |
| N-acetyltryptophan | -7.767 | 2.050E-12 | 2.000E-10 | 0.548 | 2.306 |
| N-acetyltyrosine | -8.098 | 3.505E-13 | 3.423E-11 | 0.574 | 2.634 |
| Norepinephrine | -7.270 | 2.920E-11 | 2.140E-09 | 0.615 | 2.207 |
| Ortho-hydroxyphenylacetic acid | -6.064 | 1.330E-08 | 5.360E-07 | 0.637 | 2.185 |
| Ricinoleic acid | -3.620 | 4.194E-04 | 2.560E-03 | 0.665 | 1.380 |
| Salicyluric acid | -4.773 | 4.780E-06 | 5.190E-05 | 0.025 | 3.480 |
| Sucrose | 2.646 | 9.147E-03 | 3.116E-02 | 1.776 | 1.388 |
| Taurochenodeoxycholic acid | -2.517 | 1.306E-02 | 4.160E-02 | 0.545 | 1.473 |
| Taurodeoxycholic acid | -2.523 | 1.284E-02 | 4.134E-02 | 0.545 | 1.469 |
| Trimethylamine-n-oxide | 3.744 | 2.706E-04 | 1.723E-03 | 1.928 | 1.926 |
| Tryptophanamide | -5.937 | 2.460E-08 | 7.200E-07 | 0.569 | 2.316 |
| Valerylcarnitine | -3.510 | 6.147E-04 | 3.532E-03 | 0.655 | 1.549 |
| Vanillin | -5.047 | 1.470E-06 | 2.390E-05 | 0.2705 | 3.224 |

**Note:** FC, fold change; VIP, variable importance in projection; FDR, false discovery rate; Significant differences were determined by Student's t-test with FDR correction, VIP scores derived from PLS-DA (Partial Least Squares Discriminant Analysis), and fold changes expressed as the ratio of mean metabolite concentrations between groups. Criteria for significant differences included FDR < 0.05, VIP > 1, and FC > 1.5 or < 0.7.

**Supplementary Table 3. Forty-six differentially expressed metabolites identified in the comparison between baseline and post-iTBS treatment in adolescents with depression.**

| **Metabolite Name** | ***t*** | ***p*-value** | **FDR** | **FC** | **VIP** |
| --- | --- | --- | --- | --- | --- |
| 10Z-Heptadecenoic acid | -5.831 | 3.550E-07 | 5.480E-06 | 0.402 | 1.821 |
| 3-(4-Hydroxyphenyl)propionic acid | 4.185 | 1.102E-04 | 5.872E-04 | 2.544 | 1.433 |
| 3-Hydroxybutyric acid | -6.167 | 1.049E-07 | 2.190E-06 | 0.345 | 1.895 |
| 3-Hydroxymethylglutaric acid | -7.758 | 3.078E-10 | 2.250E-08 | 0.361 | 2.504 |
| 3-Indolepropionic acid | 4.431 | 4.862E-05 | 3.097E-04 | 3.253 | 1.441 |
| 3-Methylhistidine | 3.099 | 3.131E-03 | 1.133E-02 | 1.692 | 1.223 |
| 7Z,10Z,13Z,16Z-Docosatetraenoic acid | -7.206 | 2.320E-09 | 9.730E-08 | 0.535 | 2.080 |
| Adenosine 2',3'-cyclic phosphate | -2.970 | 4.502E-03 | 1.472E-02 | 0.622 | 1.007 |
| Adenosine-5'-triphosphate | 4.640 | 2.400E-05 | 1.800E-04 | 1.578 | 1.563 |
| alpha-Linolenic acid | -4.453 | 4.510E-05 | 2.935E-04 | 0.343 | 1.757 |
| Conjugated linoleic acids | -4.565 | 3.100E-05 | 2.159E-04 | 0.638 | 1.504 |
| Decanoylcarnitine | -3.354 | 1.491E-03 | 5.903E-03 | 0.637 | 1.065 |
| D-fructose | 4.762 | 1.570E-05 | 1.281E-04 | 2.416 | 1.817 |
| Dihomo-gamma-linolenic acid | -4.282 | 7.980E-05 | 4.679E-04 | 0.511 | 1.434 |
| Elaidic acid | -4.129 | 1.325E-04 | 6.752E-04 | 0.627 | 1.500 |
| gamma-Linolenic acid | -2.709 | 9.105E-03 | 2.616E-02 | 0.660 | 1.049 |
| Glycerol 3-phosphate | 4.119 | 1.368E-04 | 6.792E-04 | 1.602 | 1.450 |
| Glycochenodeoxycholic acid | 4.349 | 6.390E-05 | 3.903E-04 | 2.010 | 1.324 |
| Glycocholic acid | 4.506 | 3.780E-05 | 2.515E-04 | 2.048 | 1.406 |
| Glycodeoxycholic acid | 4.572 | 3.020E-05 | 2.156E-04 | 2.134 | 1.394 |
| Hippuric acid | 3.214 | 2.251E-03 | 8.565E-03 | 1.807 | 1.059 |
| Indole-3-carboxaldehyde | 4.066 | 1.626E-04 | 7.810E-04 | 1.816 | 1.320 |
| Indoleacetic acid | 5.002 | 6.835E-06 | 6.260E-05 | 1.711 | 1.657 |
| Isolithocholic acid | -5.793 | 4.080E-07 | 5.800E-06 | 0.493 | 1.722 |
| Isovalerylcarnitine | 3.914 | 2.657E-04 | 1.216E-03 | 1.594 | 1.097 |
| Ketoleucine | -5.504 | 1.153E-06 | 1.350E-05 | 0.497 | 1.830 |
| Linoelaidic acid | -4.213 | 1.006E-04 | 5.459E-04 | 0.645 | 1.380 |
| Linoleic acid | -4.238 | 9.260E-05 | 5.220E-04 | 0.643 | 1.388 |
| Malonic acid | -8.745 | 8.632E-12 | 1.260E-09 | 0.180 | 2.643 |
| N2,N2-dimethylguanosine | 7.206 | 2.320E-09 | 9.730E-08 | 1.743 | 2.332 |
| N-acetylphenylalanine | 5.872 | 3.062E-07 | 4.990E-06 | 3.039 | 2.012 |
| Norepinephrine | 5.520 | 1.090E-06 | 1.330E-05 | 1.607 | 2.017 |
| Octanoylcarnitine | -3.019 | 3.929E-03 | 1.308E-02 | 0.484 | 1.125 |
| Oleic acid | -2.937 | 4.922E-03 | 1.568E-02 | 0.635 | 1.086 |
| Ortho-hydroxyphenylacetic acid | 7.024 | 4.536E-09 | 1.660E-07 | 1.840 | 2.095 |
| Palmitelaidic acid | -4.683 | 2.060E-05 | 1.592E-04 | 0.334 | 1.542 |
| Palmitoleic acid | -5.788 | 4.160E-07 | 5.800E-06 | 0.492 | 1.835 |
| Pterin | -6.281 | 6.900E-08 | 1.560E-06 | 0.543 | 2.017 |
| Suberic acid | 3.079 | 3.315E-03 | 1.160E-02 | 1.502 | 1.031 |
| Taurochenodeoxycholic acid | 5.988 | 2.010E-07 | 3.660E-06 | 2.142 | 1.689 |
| Taurocholic acid | 4.835 | 1.220E-05 | 1.025E-04 | 1.586 | 1.347 |
| Taurodeoxycholic acid | 6.119 | 1.247E-07 | 2.440E-06 | 2.147 | 1.693 |
| Tryptophanamide | 6.433 | 3.968E-08 | 1.060E-06 | 2.086 | 1.958 |
| Valerylcarnitine | 3.512 | 9.283E-04 | 3.831E-03 | 1.536 | 1.035 |
| Vitamin B1 | 6.624 | 1.970E-08 | 5.770E-07 | 3.397 | 1.926 |
| Xanthine | -7.594 | 5.610E-10 | 3.290E-08 | 0.548 | 2.521 |

**Note:** FC, fold change; VIP, variable importance in projection; FDR, false discovery rate; Significant differences were determined by Student's t-test with FDR correction, VIP scores derived from PLS-DA (Partial Least Squares Discriminant Analysis), and fold changes expressed as the ratio of mean metabolite concentrations between groups. Criteria for significant differences included FDR < 0.05, VIP > 1, and FC > 1.5 or < 0.7.
